# NMR resonance assignments of the FinO-domain of the RNA chaperone RocC

**DOI:** 10.1007/s12104-020-09983-2

**Published:** 2020-11-11

**Authors:** Reiner Eidelpes, Hyeong Jin Kim, J. N. Mark Glover, Martin Tollinger

**Affiliations:** 1 Institute of Organic Chemistry, Center for Molecular Biosciences Innsbruck (CMBI), University of Innsbruck, Innrain 80/82, 6020 Innsbruck, Austria; 2 Department of Biochemistry, University of Alberta, T6G 2H7 Edmonton, AB Canada

**Keywords:** NMR resonance assignment, Protein, Ribonucleic acid, Chaperone, Base-pairing

## Abstract

In prokaryotic species, gene expression is commonly regulated by small, non-coding RNAs (sRNAs). In the gram-negative bacterium *Legionella pneumophila*, the regulatory, trans-acting sRNA molecule RocR base pairs with a complementary sequence in the 5’-untranslated region of mRNAs encoding for proteins in the bacterial DNA uptake system, thereby controlling natural competence. Sense-antisense duplexing of RocR with targeted mRNAs is mediated by the recently described RNA chaperone RocC. RocC contains a 12 kDa FinO-domain, which acts as sRNA binding platform, along with an extended C-terminal segment that is predicted to be mostly disordered but appears to be required for repression of bacterial competence. In this work we assigned backbone and side chain ^1^H, ^13^C, and ^15^N chemical shifts of RocC’s FinO-domain by solution NMR spectroscopy. The chemical shift data for this protein indicate a mixed α/β fold that is reminiscent of FinO from *Escherichia coli*. Our NMR resonance assignments provide the basis for a comprehensive analysis of RocC’s chaperoning mechanism on a structural level.

## Biological context

The formation of molecular interactions in RNA-based regulatory processes often requires chaperoning by proteins (Woodson et al. 2018). In prokaryotic species, RNA chaperones play a particularly critical role in the regulation of gene expression. The protein RocC (repressor of competence chaperone) from the bacterium *Legionella pneumophila*, which is involved in post-transcriptional gene regulation, is such an RNA chaperone (Attaiech et al. 2016). RocC specifically binds to a non-coding 66-nucleotide RNA molecule, RocR, which in turn recognizes and binds to the 5’-untranslated region of mRNAs that encode for a variety of proteins in the bacterial DNA uptake system, such as ComEA, ComEC and ComF, controlling their expression (Attaiech et al. 2016). Within this scheme, RocC acts as an RNA chaperone by promoting strand exchange and intermolecular base pairing between the regulatory sRNA molecule RocR and its target mRNAs. Intriguingly, RocC binds to a polyU overhang at the 3’-tail of RocR, while sense-antisense base pairing occurs in a stem loop at the 5’-end of RocR, which contains a 6-nucleotide antisense sequence that binds to the complementary “RocR-box” in mRNAs. In addition, RocC binding appears to protect RocR from degradation and maintain its steady-state level. Mechanistic details of how exactly RocC enhances the formation of intermolecular base pairs and stabilizes RocR are not known to date.

RocC is a member of the FinO family of RNA chaperones, which is widespread throughout bacterial species (Attaiech et al. 2017; Glover et al. 2015; Olejniczak and Storz 2017). Despite their common occurrence, high-resolution structural data of FinO proteins are relatively rare (Chaulk et al. 2010; Ghetu et al. 2000; Gonzalez et al. 2017). The crystal structure of FinO from *E. coli* in the absence of RNA revealed a mixed α/β fold, where five helices α2–α6 pack together with four short antiparallel β-strands (β1–β4) to form a compact protein core, while an extended N-terminal helix α1 protrudes into the surrounding solvent (Ghetu et al. 2000). Two charged patches of positively charged residues in FinO were identified as likely RNA binding sites near the N-terminal end of helix α1 and on the surface of the central protein core, respectively. By combining protein-RNA crosslinking data with fluorescence resonance energy transfer, FRET (Ghetu et al. 2002), and small-angle X-ray scattering, SAXS, a low-resolution model for RNA binding to FinO from *E. coli* reconciling these data was derived (Arthur et al. 2011). In spite of this work, the structural mechanism by which these chaperones bind their RNAs and carry out their chaperone activities are poorly understood.

Here we present the solution NMR backbone and side-chain assignments of the FinO-domain of the RNA chaperone RocC. RocC from *L. pneumophila* and FinO from *E. coli* share a sequence identity of 29.1%. Major structural differences between these two chaperones include an an extended C-terminal segment spanning ca. 100 residues (127–230) in RocC, which is predicted to be mostly disordered but appears to be required for function. RocC’s FinO-domain, which is studied in this work, is located between residues 24 and 126 and acts as the major sRNA binding platform in this chaperone.

## Methods and experiments

### Sample preparation

Transformation of codon-optimized plasmids of RocC residues 24–126 from the bacterium *Legionella pneumophila* (GenBank protein code CAH11296.1), cloned in the expression vector pGEX-6P-1 containing an N-terminal GST tag, was conducted in the *E. coli* strain BL21(DE3). A 100 mL culture of Luria Bertani (LB) medium with 100 µg/mL ampicillin was inoculated with one bacterial colony and incubated overnight at 37 °C and 200 rpm. A portion of the overnight culture was centrifuged at 2000×*g* and resuspended in 1 L of M9 minimal medium (containing 1 g/L ^15^NH_4_Cl or 3 g/L ^13^C_6_-D-glucose and 1 g/L ^15^NH_4_Cl, both Cambridge Isotope Laboratories, and 100 µg/mL ampicillin) to yield a cell density of 0.1. The culture was incubated at 37 °C and 200 rpm until the cell density reached 0.5–0.6 (at 600 nm). One hour prior to induction the temperature was lowered to 18 °C. Protein expression was induced by addition of isopropyl-β-D-1-thiogalactopyranoside (IPTG, 300 mM) overnight. Cells were harvested at 3440×*g* and 4 °C for 40 min and stored at −20 °C until use. The pellets of 1 L expression medium were thawed and resuspended in 100 mL of a buffer containing 50 mM HEPES pH 7.3, 500 mM NaCl and 5% glycerol. After the addition of 200 µL protease inhibitor cocktail His-tag (Carl Roth) per 100 mL suspension, the cells were passed through a French Press and centrifuged at 15,000×*g* and 4 °C for 1 hour. The cleared lysate was loaded onto a GST affinity column (GST Trap FF 5 mL, GE Healthcare) and RocC_24−126_ was eluted with 20 mM glutathione in the same buffer at a flow rate of 2 mL/min. RocC_24−126_ containing fractions were collected and the buffer was exchanged by concentrating the sample four times to ca. 2 ml by centrifugation (Amicon Ultra 3 kDa MWCO, Merck Millipore) and refilling the tube to 7.5 mL with buffer containing 25 mM HEPES pH 7.3, 150 mM NaCl. Removal of the GST affinity tag was performed with PreScission protease (GE Healthcare). The sample was incubated with 40 units of protease and shaked gently on a nutator at 4 °C overnight. For the final purification step the protein sample was loaded onto a size exclusion column (HiLoad 16/600 Superdex 75 prep grade, GE Healthcare) and eluted isocratically at 1 mL/min, 4 °C with 25 mM HEPES pH 7.3 buffer containing 150 mM NaCl. All purification steps were monitored by SDS-PAGE gel electrophoresis with 15% gels. RocC_24−126_ samples were supplemented with 10% D_2_O (v/v) for NMR spectroscopy, with concentrations of 0.2–0.5 mM for ^15^N labeled and ^15^N/^13^C labeled protein. Mass spectrometry of unlabeled RocC_24−126_ confirmed the integrity of the protein.

### NMR spectroscopy

NMR spectra were recorded at 25 °C on a 700 MHz Bruker Avance Neo spectrometer equipped with room temperature or cryogenically cooled probes and a 500 MHz Agilent DirectDrive 2 spectrometer equipped with a room temperature probe. Backbone resonance assignments of RocC_24−126_ were performed using a two-dimensional ^1^H-^15^N-HSQC and three-dimensional HNCACB, CBCA(CO)NH, and HNCO experiments. A two-dimensional ^1^H-^13^C-HSQC and three-dimensional (H)CC(CO)NH-TOCSY, H(CCO)NH-TOCSY, ^1^H-^15^N-TOCSY-HSQC, ^1^H-^15^N-NOESY-HSQC, and ^1^H-^13^C-NOESY-HSQC experiments were used for side-chain assignments (Sattler et al. 1999). Data processing was performed with NMRPipe (Delaglio et al. 1995) and the CcpNMR software package was used for resonance assignment (Vranken et al. 2005).

### Assignments and data deposition

**Fig. 1:**
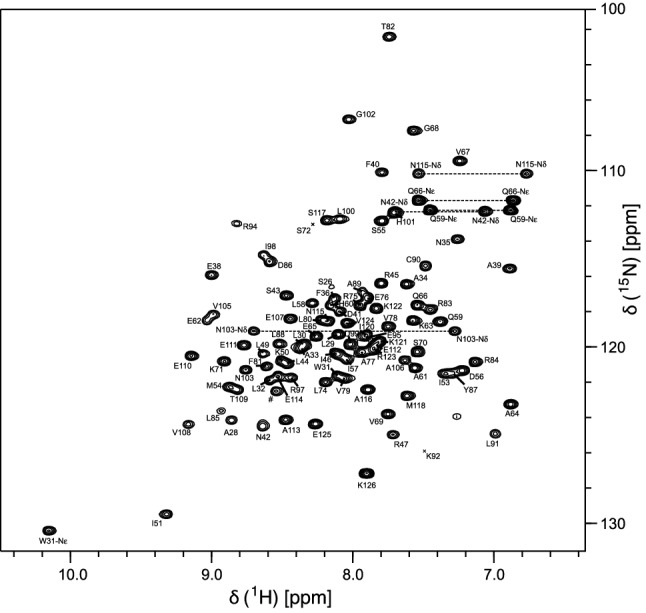
700 MHz ^1^H-^15^N-HSQC spectrum of RocC (residues 24–126) in 25 mM HEPES buffer, pH 7.3, 150 mM NaCl, supplemented with 10% D_2_O at 25 °C. Resonance assignments are shown using single letter codes and horizontal lines indicate asparagine and glutamine NH_2_ side-chain resonances. Asterisks are used to mark positions of residues below the intensity cut-off and hashtags indicate resonances deriving from the N-terminal PreScission cleavage site. Resonance assignments are available online at the BMRB repository (accession number 50404)

The ^1^H-^15^N-HSQC spectrum of RocC_24−126_ (Fig. 1) shows that backbone amide resonances are well dispersed, as expected for a folded protein. We obtained assignment of the backbone amide resonances for 92 of 100 non-proline residues in RocC_24−126_. For a number of residues at the N-terminus of the protein (Ala24, Arg25, Asp27), as well as Gly52, Lys73, Ala93, Val96 and Lys119, assignmens of backbone amide resonances were not obtained. Backbone C^α^ and carbonyl C’ resonances were assigned for 102 and 93 out of 103 residues, corresponding to 99% and 90% completeness, respectively. Side-chain C^β^, C^γ^ and C^δ^ resonance assignments are 99%, 78% and 78% complete, while ^1^H resonances could be assigned for 92.9% of H^α^ nuclei along with 42.9%, 22.7% and 23.3% of aliphatic side-chain H^β^, H^γ^ and H^δ^ resonances, respectively. Assignments of the side-chain amides (^1^H and ^15^N) were obtained for three asparagines and both glutamines.

**Fig. 2:**
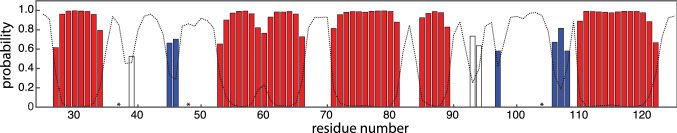
Secondary structure of RocC (residues 24–126) as predicted by TALOS+, based on backbone H^N^, N, C’, C^α^ and C^β^ chemical shifts. Secondary structure probabilities are shown as bars (*red*, α-helices; *blue*, β-strands); short segments of helix-like backbone geometry covering up to two amino acid residues are indicated by open bars and loop probabilities are shown as dotted line (*black*). The positions of prolines are denoted by *asterisks*

Based on the H^N^, N, C’, C^α^ and C^β^ chemical shifts, the TALOS + software (Shen et al. 2009) was used to predict the secondary structure elements of RocC_24−126_ (Fig. 2). These data indicate the presence of a mixed α/β fold, in accordance with FinO from *E. coli* (Ghetu et al. 2000) and the ProQ homolog Lpp 1663 from *Legionella pneumophila* (Immer et al. 2018). At least three short β-strands are present in RocC_24−126_, along with four α-helices of five or more amino acid residues in length. For the central helix between residues Ile53 and Gln66 the NMR chemical shift data indicate a possible break or kink around position 60. In addition, moderate propensities for local helix-like conformation exist for residues Arg94 and Glu95, as well as for alanine at position 39. For Ala39, however, the probability to have local helical geometry barely exceeds 50%, while the probability for a local loop structure is just below this threshold.

The NMR chemical shift assignments of RocC_24−126_ have been deposited at the Biological Magnetic Resonance Data Bank (http://www.bmrb.wisc.edu) with the BMRB accession number 50404. These data will enable us to characterize mechanistic details of the RocC-RocR chaperoning system in future studies.

## References

[CR1] Arthur DC, Edwards RA, Tsutakawa S, Tainer JA, Frost LS, Glover JN (2011). Mapping interactions between the RNA chaperone FinO and its RNA targets. Nucl Acids Res.

[CR2] Attaiech L (2016). Silencing of natural transformation by an RNA chaperone and a multitarget small RNA. Proc Natl Acad Sci USA.

[CR3] Attaiech L, Glover JNM, Charpentier X (2017). RNA chaperones step out of Hfq’s shadow. Trends Microbiol.

[CR4] Chaulk S (2010). N. meningitidis 1681 is a member of the FinO family of RNA chaperones. RNA Biol.

[CR5] Delaglio F, Grzesiek S, Vuister GW, Zhu G, Pfeifer J, Bax A (1995). NMRPipe: a multidimensional spectral processing system based on UNIX pipes. J Biomol NMR.

[CR6] Ghetu AF, Gubbins MJ, Frost LS, Glover JN (2000). Crystal structure of the bacterial conjugation repressor FinO. Nat Struct Biol.

[CR7] Ghetu AF, Arthur DC, Kerppola TK, Glover JN (2002). Probing FinO-FinP RNA interactions by site-directed protein-RNA crosslinking and gelFRET. RNA.

[CR8] Glover JNM, Chaulk SG, Edwards RA, Arthur D, Lu J, Frost LS (2015). The FinO family of bacterial RNA chaperones. Plasmid.

[CR9] Gonzalez GM (2017). Structure of the Escherichia coli ProQ RNA-binding protein. RNA.

[CR10] Immer C, Hacker C, Woehnert J (2018). NMR resonance assignments for a ProQ homolog from Legionella pneumophila. Biomol NMR Assign.

[CR11] Olejniczak M, Storz G (2017). ProQ/FinO-domain proteins: another ubiquitous family of RNA matchmakers?. Mol Microbiol.

[CR12] Sattler M, Schleucher J, Griesinger C (1999). Heteronuclear multidimensional NMR experiments for the structure determination of proteins in solution. Prog Nucl Magn Reson Spectrosc.

[CR13] Shen Y, Delaglio F, Cornilescu G, Bax A (2009). TALOS+: a hybrid method for predicting protein backbone torsion angles from NMR chemical shifts. J Biomol NMR.

[CR14] Vranken WF (2005). The CCPN data model for NMR spectroscopy: development of a software pipeline. Proteins.

[CR15] Woodson SA, Panja S, Santiago-Frangos A (2018). Proteins that chaperone RNA regulation. Microbiol Spectr.

